# Correction: Evaluation of the therapeutic efficiency and efficacy of blood purification in the treatment of severe acute pancreatitis

**DOI:** 10.1371/journal.pone.0332933

**Published:** 2025-09-22

**Authors:** Hongwei Huang, Zhongshi Huang, Menghua Chen, Ken Okamoto

An additional affiliation is missing for the first author. Hongwei Huang is also affiliated with the Emergency and Intensive Care Unit, Juntendo University Urayasu Hospital, Urayasu, Japan.

## References

[1] Huang H, Huang Z, Chen M, Okamoto K. Evaluation of the therapeutic efficiency and efficacy of blood purification in the treatment of severe acute pancreatitis. PLoS One. 2024;19(1):e0296641. doi: 10.1371/journal.pone.0296641 38181043 PMC10769011

