# Supplementary material for: Evaluation of the therapeutic efficiency and efficacy of blood purification in the treatment of severe acute pancreatitis
Source: PLoS One. 2024 Jan 5;19(1):e0296641. doi: 10.1371/journal.pone.0296641 (PMC10769011; doi:10.1371/journal.pone.0296641)
Supplement: Attachment — Submitted filename: review.docx [file pone.0296641.s001.docx]

1.Is the unit of creatinine level “mmol/L” or“umol/L”

2.Please add the cause of pancreatitis like “Gallstones,Alcohol abuse,Hyperlipidemia” in the baseline character.

3.In table 3 there was statistically significant difference in 1-year all-cause mortality between two groups(P=0.04).What is the 1-year survival curves between two groups?

4 The English in this paper should be improved. I recommend the authors have it checked by a native speaker of English familiar with this field.
